# Cultural appropriateness, co-creation, and subjective vitality: a pre–post structural modeling study of immersive performances in rural Guangdong, China

**DOI:** 10.3389/fpubh.2026.1945573

**Published:** 2026-09-18

**Authors:** Yuqi Shang

**Affiliations:** Zhujiang College, South China of Agricultural University, Guangzhou, China

**Keywords:** cultural appropriateness, immersive performances, performance co-creation, social connectedness, subjective vitality

## Abstract

**Background:**

This study examined short-term changes in social connectedness and subjective vitality following culturally adapted, co-created immersive performances, and explored baseline-adjusted associations among cultural appropriateness, co-creation engagement, social connectedness, and vitality.

**Methods:**

A single-group pre–post field study was conducted across four communities in Guangdong, China. Data from 786 matched participants were analyzed using paired-samples tests and structural equation modeling with bootstrap estimation.

**Results:**

Social connectedness was higher after participation than at baseline (3.48 vs. 3.27, *d*z = 0.40, *p* < 0.001), as was subjective vitality (4.57 vs. 4.20, *d*z = 0.46, *p* < 0.001). Cultural appropriateness was positively associated with co-creation engagement (*β* = 0.551), which was associated with social connectedness (*β* = 0.355); social connectedness was associated with vitality (*β* = 0.419). Significant indirect associations were observed through co-creation alone (*β* = 0.068, 95% CI [0.037, 0.102]) and through co-creation and social connectedness (*β* = 0.082, 95% CI [0.065, 0.100]).

**Conclusion:**

Participants reported higher immediate social connectedness and subjective vitality after the performances, while the structural findings were consistent with theory-informed indirect pathways. Given the study design, these associations should not be interpreted as evidence of causal mediation.

## 1 Introduction

Public health research increasingly recognizes that health and psychosocial functioning are shaped not only by clinical care but also by social relationships, cultural participation, and access to community resources. Social prescribing reflects this broader understanding by connecting individuals with non-clinical activities and resources, including community groups, cultural organizations, physical activities, and nature-based programmes (1–5). Within this field, arts-based prescribing and community arts participation have received growing attention because artistic activities can provide opportunities for emotional expression, social interaction, meaning-making, and community participation (6). Research on music, visual arts, dance, theatre, museums, and other community-based creative activities has reported associations with psychological wellbeing, reduced social isolation, and stronger social participation across different populations and settings (7–14).

However, the evidence remains heterogeneous because arts-based programmes differ substantially in artistic form, duration, intensity, participant characteristics, implementation context, and degree of active participation. Consequently, it is increasingly important to move beyond asking only whether arts participation is associated with positive psychosocial outcomes and to examine how programme characteristics, participant experiences, and social processes are related to these outcomes. The INNATE framework, for example, emphasizes that arts-in-health activities should not be understood as standardized exposures whose consequences occur automatically; rather, their outcomes are shaped by interactions among programme components, participant experiences, and contextual conditions (15). This perspective places particular importance on the ways in which artistic programmes are designed, experienced, and embedded within local communities.

One potentially important programme characteristic is cultural appropriateness. Cultural appropriateness refers to the extent to which the content, mode of delivery, communication practices, and setting of an intervention are consistent with participants’ cultural values, everyday experiences, and community needs (16). More broadly, recent cross-cultural evidence indicates that culturally rooted value systems can shape how individuals interpret and respond to the same behavioral context (17). In community arts settings, cultural appropriateness extends beyond the superficial inclusion of culturally recognizable symbols. It may involve the respectful and meaningful incorporation of local stories, dialects, traditional objects, collective memories, familiar social roles, and culturally significant spaces. Related research on Southern Han Chinese ritual practices similarly suggests that cultural meanings are locally embedded and may vary with regional language, family traditions, and urban–rural context (18). Such elements can provide participants with recognizable cultural resources through which they interpret and contribute to an artistic experience. In rural communities in particular, shared cultural materials may also serve as resources for collective identity by connecting residents to common histories, places, and forms of belonging.

Immersive performance provides a particularly suitable context in which to examine these processes. Unlike conventional performance formats based primarily on passive spectatorship, immersive performances invite participants to move through performance spaces, assume roles, interact with performers, complete narrative tasks, and contribute to the development of the artistic experience (19, 20). When these activities are embedded in community settings, participants may contribute local stories and memories, use dialect expressions, recognize traditional objects, create sounds and movements, and participate in collective narrative decisions. These characteristics blur the conventional distinction between performer and audience and create opportunities for what can be described as performance co-creation engagement. Co-creation theory proposes that value is generated through interaction and the integration of resources contributed by both providers and participants rather than being delivered unilaterally by programme organizers (21–23). From this perspective, culturally recognizable and locally meaningful materials may provide participants with knowledge and experiential resources that are associated with greater willingness to contribute to the performance.

Co-created participation may also be relevant to social connectedness. Social connectedness refers to the subjective experience of belonging, interpersonal closeness, acceptance, and inclusion within a social group or community (24). Strong social relationships are consistently associated with better psychological and physical health, whereas social isolation and weak social integration represent important public-health concerns (25, 26). Participatory arts activities can create opportunities for shared attention, interpersonal exchange, coordinated action, self-disclosure, and the joint production of meaningful experiences. A social identity perspective further suggests that shared roles, culturally recognizable symbols, and coordinated group activity may increase the salience of a shared sense of “we,” thereby providing a plausible basis for rapid feelings of closeness and belonging (27, 28). Research on coordinated singing, music, and movement similarly indicates that shared activity can facilitate relatively rapid social bonding and perceived interpersonal connection (29, 30).

The use of shared cultural memories may be particularly relevant in communities containing participants from different generations. Older residents may contribute local knowledge, memories, dialect expressions, and interpretations of community traditions, while younger residents may encounter, reinterpret, and enact these resources through role-taking and collaborative performance. In this sense, cultural memory is not merely presented to participants as heritage content but can become a jointly enacted social resource. Such intergenerational exchange may enable older community members to participate as cultural knowledge holders while allowing younger participants to engage actively with local histories. Although the present study does not directly test intergenerational transmission as a separate mechanism, this perspective provides an additional theoretical rationale for examining cultural appropriateness, co-creation, and social connectedness together within community-based immersive performance.

Alongside social connectedness, the present study examines subjective vitality as an immediate state-like psychosocial outcome. Subjective vitality refers to the experience of feeling energetic, alert, and psychologically alive (31). It is particularly suitable for a brief pre–post field study because it captures participants’ current psychological energy rather than asking them to evaluate broader or more enduring dimensions of mental health. Subjective vitality should therefore be distinguished from general psychological wellbeing or public mental health, which represent wider and more multidimensional constructs. Physical movement, sensory stimulation, emotional engagement, and interpersonal interaction during immersive performance provide plausible reasons why vitality may be higher immediately after participation (32). At the same time, participants who report stronger post-performance social connectedness may also report greater vitality. However, because these experiences can occur and be assessed within the same immediate post-performance period, their temporal ordering cannot be assumed solely from statistical associations.

These issues have particular relevance in rural Chinese community settings. Formalized social-prescribing models based on clinical referral and dedicated link workers should not be assumed to operate uniformly across rural communities. Instead, locally embedded community structures may play an important role in connecting residents with non-clinical cultural and social resources. Village committees can facilitate resident mobilization, communication, and coordination of local resources; rural cultural stations and local cultural organizations can provide venues, cultural programmes, and access to community cultural resources; and local cultural knowledge holders may contribute cultural legitimacy, local memory, and intergenerational knowledge. Rather than constituting a formal clinical referral system, these locally embedded actors can be conceptualized as elements of an informal referral architecture through which residents may become connected to community-based cultural participation. This perspective is consistent with the broader logic of social prescribing, in which non-clinical community resources can complement conventional health and social services by creating opportunities for social participation and connection (3, 4). In the present study, recruitment and implementation were supported by community committees, local cultural organizations, and resident communication networks, illustrating the practical relevance of community-based organizational infrastructures for making such activities accessible.

Despite increasing interest in arts participation, social prescribing, and community wellbeing, several gaps remain. First, much of the existing evidence on arts-based health promotion has focused on Western or urban contexts, while comparatively less is known about culturally embedded participatory performance in rural Chinese communities. Second, existing studies often evaluate arts participation as a broad exposure, providing less information about how participants’ perceptions of cultural appropriateness, their degree of co-creative engagement, and their sense of social connectedness are related within the same community arts experience. Third, much of the existing evidence linking programme perceptions, co-creation, and psychosocial outcomes is cross-sectional. A pre–post design can provide information about short-term within-participant changes in outcomes such as social connectedness and subjective vitality while allowing baseline levels to be incorporated into subsequent structural analyses.

At the same time, the inferential limits of such a design need to be clearly recognized. A single-group pre–post design without a comparison condition cannot establish that observed changes were caused exclusively by the immersive performance. Moreover, perceived cultural appropriateness, performance co-creation engagement, post-performance social connectedness, and post-performance subjective vitality are assessed at the same post-performance occasion. Consequently, although a structural model can evaluate associations among these constructs and test theory-informed indirect pathways, statistical directionality does not establish their temporal or causal ordering. The resulting model should therefore be interpreted as a baseline-adjusted structural representation of hypothesized relationships rather than as definitive evidence of a sequential causal mechanism.

Against this background, the present study examines four culturally adapted, co-created immersive performance activities conducted in rural communities in Guangdong Province, China. The performances shared a common structure involving spatial exploration, role-based interaction, local story collection, dialect or environmental sound creation, traditional-object recognition, collective movement, and participatory decisions about narrative development, while the specific stories, spaces, dialects, and cultural materials were adapted to each community. Social connectedness and subjective vitality were measured immediately before and after participation, whereas perceived cultural appropriateness and performance co-creation engagement were assessed after the performance as evaluations of the experienced activity. The study has two principal objectives. First, it examines whether participants report short-term pre–post differences in social connectedness and subjective vitality. Second, it evaluates whether baseline-adjusted structural associations among perceived cultural appropriateness, performance co-creation engagement, post-performance social connectedness, and post-performance subjective vitality are consistent with a theory-informed indirect pathway. By distinguishing observed pre–post change from post-performance structural associations, the study aims to provide a more cautious and context-sensitive account of how culturally embedded immersive performance relates to immediate psychosocial experiences in rural community settings.

## 2 Literature review and hypotheses

### 2.1 Immersive performances and immediate psychosocial outcomes

Arts-based community activities are increasingly recognized as non-clinical resources that may support social participation and positive psychosocial experiences. Research on social prescribing and community arts suggests that engagement with music, dance, theatre, museums, and other cultural activities may help address psychosocial needs that are not fully met through conventional healthcare services (1, 2, 7, 33).

The psychosocial relevance of arts participation may depend on the characteristics of the activity itself. The INNATE framework proposes that arts-in-health outcomes are shaped by interactions among programme components, participant experiences, and contextual conditions rather than by artistic exposure alone (15). Immersive performances are particularly relevant because they combine artistic engagement with physical movement, sensory stimulation, interpersonal interaction, role-taking, and shared meaning-making. Unlike conventional performances based largely on passive spectatorship, they invite participants to move through performance spaces, interact with performers, complete narrative tasks, and contribute to the unfolding experience (19, 20).

These participatory features may be associated with immediate psychosocial changes. Shared tasks, collective storytelling, and face-to-face interaction can create opportunities for communication, mutual recognition, and interpersonal connection, while physical movement, emotional involvement, and sensory engagement may coincide with greater psychological activation. Previous studies of community arts and participatory creative activities have similarly reported associations with social engagement, perceived connection, and positive psychological experiences (8, 10, 13, 14).

In this study, subjective vitality is treated as an immediate, state-like psychosocial outcome rather than as a comprehensive indicator of general wellbeing or mental health. It refers to the experience of feeling energetic, alert, and psychologically alive (31), making it suitable for assessing short-term changes immediately following participation. Because the present study uses a single-group pre–post design without a comparison condition, the following hypotheses concern within-participant differences rather than definitive intervention effects:

*H1:* Participants will report higher social connectedness after the immersive performance than before the performance.

*H2:* Participants will report higher subjective vitality after the immersive performance than before the performance.

### 2.2 Cultural appropriateness, performance co-creation, and social connectedness

Cultural appropriateness refers to the extent to which the content, delivery, and setting of an intervention are perceived as consistent with participants’ cultural experiences, values, communication practices, and community needs (16). It extends beyond cultural authenticity because an activity may contain recognizable cultural elements without necessarily being accessible or meaningful to the intended participants. In rural immersive performances, cultural appropriateness may be reflected in the respectful use of local stories, dialects, traditional objects, community memories, and culturally significant spaces. Such locally meaningful elements may provide participants with familiar cultural resources through which they can interpret and engage with the performance.

In the present study, performance co-creation engagement refers to the extent to which participants actively contribute their knowledge, stories, actions, interactions, and decisions to the immersive experience. Co-creation theory suggests that value emerges through the integration of resources contributed by both providers and participants rather than being delivered unilaterally (21–23). Within immersive performances, co-creation may involve sharing local stories, participating in role-based dialogue, creating sounds or movements, completing narrative tasks, and contributing to collective decisions. When participants recognize their own cultural experiences within the activity, they may possess relevant knowledge and memories that can be meaningfully contributed. Perceived cultural appropriateness is therefore expected to be positively associated with stronger co-creation engagement.

Shared cultural memory may further strengthen the social relevance of co-creation across generations. Older residents may contribute lived memories, dialect expressions, local customs, and knowledge of community history, while younger participants may encounter and reinterpret these cultural resources through role-taking and collaborative performance. In this way, local cultural memory can become a jointly enacted community resource rather than simply content presented to an audience. Such participation may create opportunities for intergenerational interaction and reciprocal recognition, although the present study does not directly measure intergenerational transmission as a separate mechanism.

A social identity perspective also helps explain why co-creation may be associated with social connectedness. Social identity research suggests that shared group membership can become more salient when people participate in common activities and recognize themselves as part of a meaningful collective (27, 28). In immersive performance, shared roles, familiar cultural symbols, coordinated movement, and collective narrative tasks may strengthen a temporary sense of “we.” Role-taking reduces the distinction between passive audience members and active contributors, while coordinated action requires mutual attention and responsiveness. Research on group arts and coordinated activity similarly indicates that shared singing, movement, and collaborative participation can facilitate interpersonal closeness and rapid social bonding (8, 10, 29, 30). These processes provide a theoretical basis for expecting stronger co-creation engagement to be associated with greater post-performance social connectedness.

Because perceived cultural appropriateness and performance co-creation engagement were both assessed after the performance, the proposed ordering should be interpreted as theory-informed structural associations rather than as established temporal causation. Accordingly, the following hypotheses are proposed:

*H3:* Perceived cultural appropriateness will be positively associated with performance co-creation engagement.

*H4:* Performance co-creation engagement will be positively associated with post-performance social connectedness after controlling for baseline social connectedness.

### 2.3 Social connectedness, subjective vitality, and the hypothesized serial indirect pathway

Social connectedness reflects the subjective experience of belonging, interpersonal closeness, acceptance, and inclusion within a social group or community (24). Strong social relationships are associated with better psychological and physical health, whereas social isolation and weak social integration are associated with increased health risks (25–28). In community arts settings, shared participation, coordinated action, and interpersonal interaction may therefore provide important social resources by reinforcing recognition, inclusion, and a sense of belonging.

Social connectedness may also be positively associated with subjective vitality. Feeling accepted and connected to others may reduce interpersonal uncertainty and coincide with greater enthusiasm, motivation, and psychological energy. Related evidence from Chinese populations also suggests that perceived social support and socially oriented interaction are meaningfully associated with psychological well-being and distress (34). During immersive performances, participants who feel included in collective storytelling, shared tasks, and artistic decision-making may consequently report greater alertness and vitality after participation. This interpretation is consistent with research suggesting that the psychosocial value of community arts is often associated with social participation, shared identity, coordinated activity, and interpersonal support rather than artistic exposure alone (2, 10, 15, 28–30). Accordingly:

*H5:* Post-performance social connectedness will be positively associated with post-performance subjective vitality after controlling for baseline subjective vitality.

Performance co-creation engagement may also be directly associated with immediate subjective vitality. Co-creation involves active role participation, physical movement, attention, creative decision-making, and a sense of agency, all of which may coincide with greater psychological activation. Thus, in addition to the proposed pathway involving social connectedness, the structural model includes an additional theoretically relevant association between performance co-creation engagement and post-performance vitality. This pathway is treated as supplementary rather than as a primary hypothesis.

The proposed framework therefore distinguishes four conceptually related components: perceived cultural appropriateness as a programme-related evaluation, performance co-creation engagement as an experiential participation construct, social connectedness as a proximal social state, and subjective vitality as an immediate state-like psychosocial outcome. The theory-informed ordering proposes that cultural appropriateness is associated with greater co-creation engagement, co-creation is associated with stronger post-performance social connectedness, and social connectedness is associated with higher post-performance vitality. However, because cultural appropriateness, co-creation engagement, post-performance social connectedness, and post-performance vitality were assessed at the same post-performance occasion, this ordering should not be interpreted as established temporal or causal mediation. Rather, the model tests whether the observed associations are consistent with the proposed serial indirect pathway after adjustment for baseline social connectedness and vitality. Accordingly:

*H6:* Perceived cultural appropriateness will show a positive serial indirect association with post-performance subjective vitality through performance co-creation engagement and post-performance social connectedness, after controlling for baseline social connectedness and subjective vitality.

Taken together, the proposed framework integrates complementary perspectives on programme design, participation, and psychosocial experience. The INNATE framework emphasizes that arts-in-health outcomes are shaped by interactions among programme characteristics, participant experiences, and context (15), while cultural appropriateness and co-creation perspectives explain why locally meaningful content may be associated with greater participant contribution (16, 21–23). Social identity perspectives further suggest that shared cultural symbols, role-taking, and coordinated participation may strengthen feelings of group belonging and interpersonal connection (27, 28). Social connectedness may, in turn, be associated with greater immediate subjective vitality as a state-like psychosocial experience (31). Together, these perspectives provide a theory-informed basis for the proposed structural associations and serial indirect pathway, while not implying that the temporal or causal ordering among the post-performance constructs has been established.

The analytical framework is illustrated in Figure 1.

**Figure 1:**
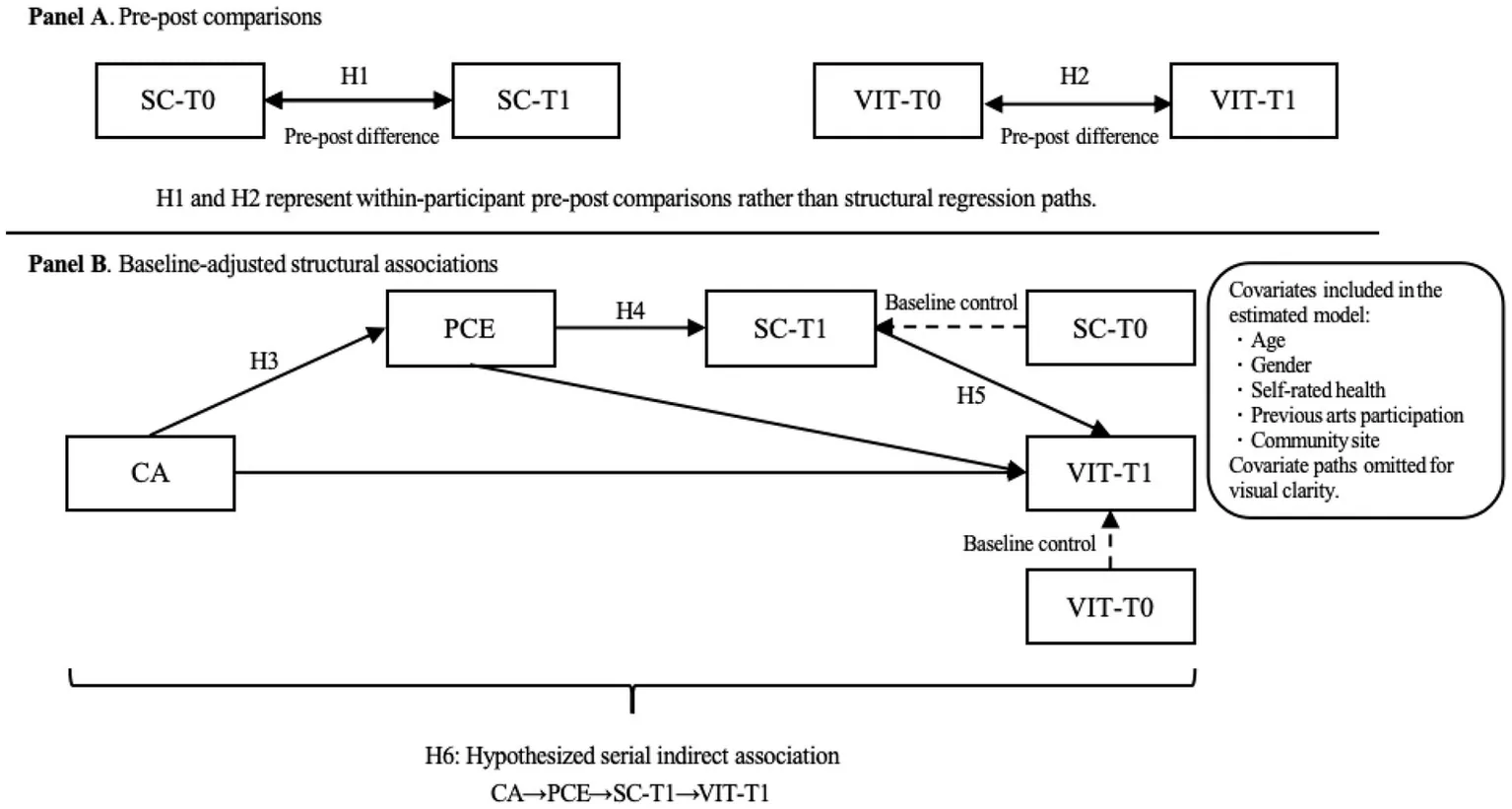
Analytical framework for pre–post comparisons and baseline-adjusted structural associations. CA, perceived cultural appropriateness; PCE, performance co-creation engagement; SC, social connectedness; VIT, subjective vitality; T0, pre-performance measurement; T1, immediate post-performance measurement. Panel **(A)** presents the within-participant pre–post comparisons corresponding to H1 and H2 and should not be interpreted as structural regression paths. Panel **(B)** presents the baseline-adjusted structural associations corresponding to H3–H5. H6 represents the hypothesized serial indirect association CA → PCE → SC-T1 → VIT-T1. Dashed arrows indicate baseline control paths. Additional associations involving post-performance vitality are shown in Panel **(B)**. Age, gender, self-rated health, previous arts participation, and community site were included as covariates but are omitted for visual clarity.

## 3 Methods

### 3.1 Data collection

A single-group pre–post field design was conducted between March and June 2026 in four communities in Panyu and Conghua Districts, Guangzhou, Guangdong Province, China. Participants were recruited through a community-based volunteer approach. Activity information was distributed through village committees, local cultural centres, and resident WeChat groups, and eligible residents volunteered to participate. Eligibility criteria included being at least 18 years old, having lived in the community for at least 6 months, being able to understand the questionnaire and activity instructions, and providing informed consent. Each community recruited 200 participants, yielding an initial sample of 800.

One immersive performance session was conducted in each community. All four sessions were delivered by the same trained performance and facilitation team and lasted approximately 100 min. A standardized core script and researcher-completed implementation checklist were used to maintain consistency across sites. Core components included spatial exploration, role-based interaction, narrative tasks, local story or dialect contributions, collective sound and movement creation, and participatory narrative decisions. No major deviations from the core protocol were recorded. Stories, objects, music, dialects, and spatial routes were adapted to each community.

Participants completed the baseline questionnaire approximately 10–15 min before the performance and the post-performance questionnaire immediately afterward. Social connectedness and subjective vitality were assessed at both time points, whereas perceived cultural appropriateness and performance co-creation engagement were assessed only after participation. Anonymous matching codes were used to link the two questionnaires.

To reduce response bias, participants completed questionnaires independently and were informed that responses were anonymous and confidential, with no right or wrong answers. They were asked not to discuss their responses, and questionnaires were collected by research assistants who were not informed of the study hypotheses. Because the post-performance assessment was administered immediately after participation, transient emotional arousal could not be fully excluded.

Of the 800 initially recruited participants, 14 were excluded: five because the pre- and post-performance questionnaires could not be matched, four because they failed one prespecified attention-check item, and five because they had one or more missing responses on the principal study measures. The final analytical sample comprised 786 participants. Ethical approval was obtained from the Ethical Commission of Zhujiang College of South China of Agricultural University (ZJSCAU-IRB2025-73). All participants provided informed consent, and the study was conducted in accordance with the Declaration of Helsinki. The participant recruitment, intervention, and questionnaire administration procedure is summarized in Figure 2.

**Figure 2:**
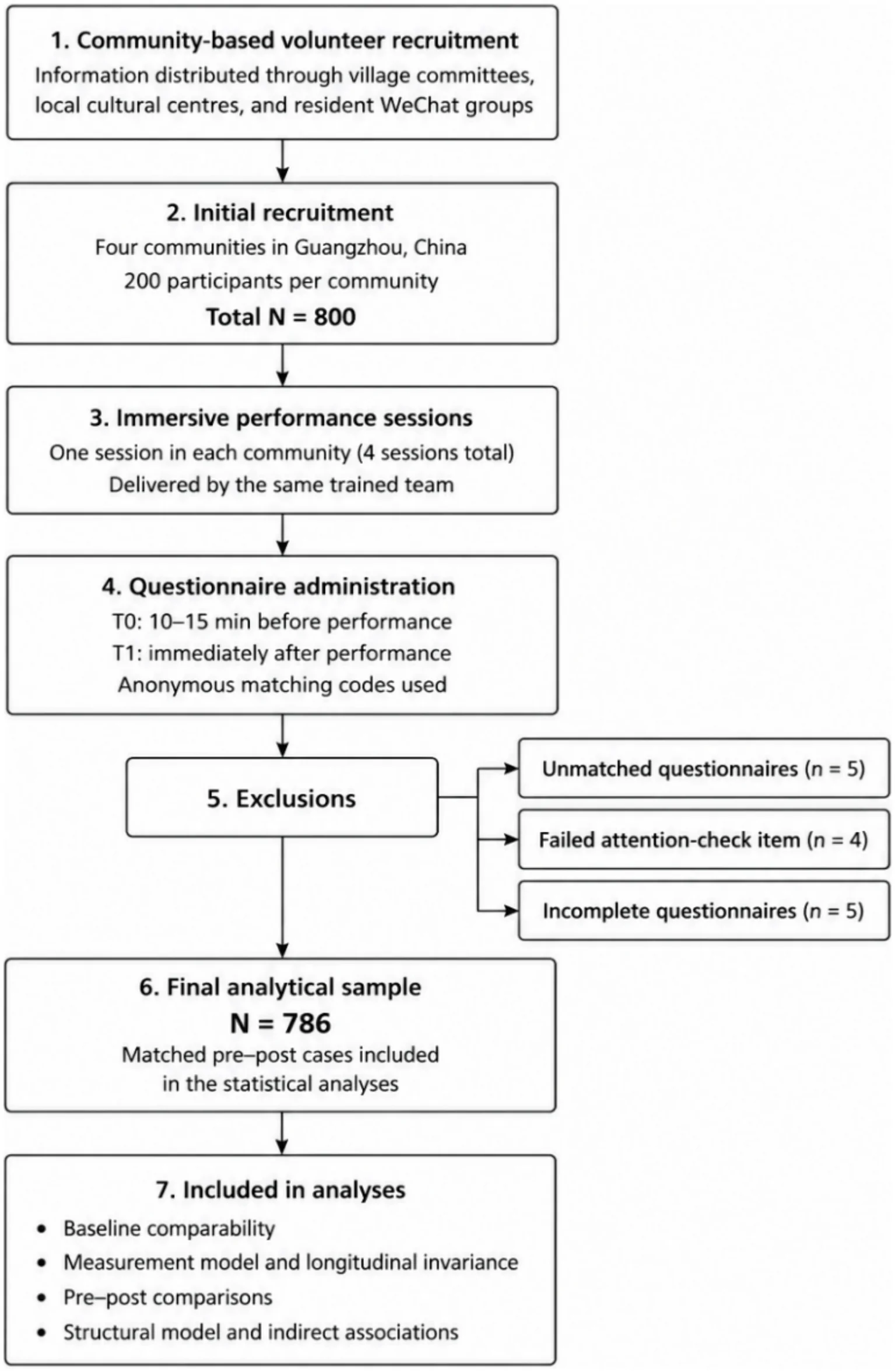
Participant flow and analytical sample construction. T0, pre-performance measurement; T1, post-performance measurement.

### 3.2 Measures

All measures were administered in Chinese. Established English-language measures were translated into Chinese and independently back-translated following standard cross-cultural translation procedures (35). Items adapted for the present study were contextualized to reflect the rural immersive-performance setting while retaining the conceptual meaning of the original constructs.

Perceived cultural appropriateness (CA) was assessed after the performance using four items informed by the cultural adaptation literature and the Intervention Appropriateness Measure (16, 36). The items assessed the perceived suitability of the performance content, cultural elements, participation format, and community setting. Performance co-creation engagement (PCE) was assessed after the performance using four items adapted from co-creation and public-service value-creation research (21–23), capturing participants’ contributions of ideas, actions, local stories, interactions, and decisions. Both constructs were rated on seven-point Likert scales ranging from 1 (strongly disagree) to 7 (strongly agree). CA and PCE were intentionally assessed only after participation because they represent evaluations of the in-situ performance experience and could not be meaningfully evaluated before exposure to the activity. However, their post-performance assessment does not establish temporal precedence among CA, PCE, post-performance social connectedness, and post-performance subjective vitality.

Social connectedness was measured before and after the performance using four items adapted from the Social Connectedness Scale (24). The items assessed perceived belonging, interpersonal connection, inclusion, and the ability to share experiences with others in the community and were rated on a five-point Likert scale. State subjective vitality was measured at both time points using five items adapted from the Subjective Vitality Scale (31), assessing immediate feelings of energy, alertness, and psychological aliveness on a seven-point scale. The same items and response formats were used at the pre- and post-performance assessments.

The structural analyses adjusted for age, gender, self-rated health, previous arts participation, and community site. Self-rated health was assessed on a five-point scale, with higher scores indicating better perceived health. Previous arts participation was assessed using a five-point frequency scale, with higher scores indicating more frequent prior participation in arts activities. Community site was included as a control variable to account for potential differences across the four performance locations.

### 3.3 Data analysis

Data were analyzed using IBM SPSS Statistics, R with the lavaan package, and SmartPLS 4. Descriptive statistics were calculated for the final sample of 786 participants. Baseline comparability across the four communities was examined using chi-square tests for age group, gender, and educational attainment, with Cramér’s V reported as an effect-size measure; continuous age was additionally compared using one-way analysis of variance.

Construct reliability and validity were assessed using Cronbach’s alpha, McDonald’s omega, composite reliability, standardized indicator loadings, average variance extracted, the Fornell–Larcker criterion, and the Heterotrait–Monotrait ratio (37–39). Longitudinal measurement invariance of social connectedness and subjective vitality was examined separately using covariance-based confirmatory factor analysis in R (lavaan) with maximum-likelihood estimation. Configural, metric, and scalar invariance were evaluated using *χ*^2^, CFI, TLI, RMSEA, and SRMR. Changes in model fit were assessed using |ΔCFI| ≤ 0.010, ΔRMSEA ≤ 0.015, and ΔSRMR ≤ 0.030 for metric invariance and ≤ 0.010 for scalar invariance (40, 41).

After adequate measurement invariance was established, H1 and H2 were tested using paired-samples t tests, with mean changes, 95% confidence intervals, and within-participant Cohen’s dz. reported (42). H3–H6 were examined using a baseline-adjusted structural model in SmartPLS 4, controlling for baseline social connectedness and vitality as well as age, gender, self-rated health, previous arts participation, and community site. Direct, total, and specific indirect associations were estimated using 5,000 bootstrap samples with bias-corrected 95% confidence intervals. The indirect analyses included both the pathway through performance co-creation alone and the hypothesized serial pathway through performance co-creation and post-performance social connectedness.

Because participants were recruited from four community-specific performances, site-specific descriptive analyses and leave-one-community-out sensitivity analyses were conducted in addition to controlling for community site. Harman’s single-factor test was retained only as a descriptive diagnostic of common-method variance and was not interpreted as evidence that common-method bias had been eliminated (43). Given the single-group design and concurrent post-performance measurement of the principal experiential constructs, structural findings were interpreted as baseline-adjusted associations rather than causal or temporally established mediation.

## 4 Results

### 4.1 Participant characteristics and preliminary assessment

A total of 800 participants were initially recruited from the four communities. Fourteen participants were excluded from the paired analytical sample: five because their pre- and post-performance questionnaires could not be matched, four because they failed the prespecified attention-check item, and five because they had missing responses on one or more principal study measures. The final analytical sample therefore comprised 786 participants (98.3%). The four community samples were similar in size, with 196 participants from Panyu Community 1, 197 from Panyu Community 2, 196 from Conghua Community 1, and 197 from Conghua Community 2. Participants had a mean age of 48.00 years (SD = 12.88), and 58.3% were female. Participant characteristics are summarized in Table 1.

**Table 1:** Participant characteristics (*N* = 786).

Characteristic	Category/Statistic	*n* (%) or *M* (SD)
Community	Panyu Community 1	196 (24.9%)
	Panyu Community 2	197 (25.1%)
	Conghua Community 1	196 (24.9%)
	Conghua Community 2	197 (25.1%)
Age, years	Mean (SD)	48.00 (12.88)
Age group	18–29 years	57 (7.3%)
	30–44 years	263 (33.5%)
	45–59 years	318 (40.5%)
	≥60 years	148 (18.8%)
Gender	Female	458 (58.3%)
	Male	328 (41.7%)
Education	Primary school or below	127 (16.2%)
	Junior secondary school	243 (30.9%)
	Senior secondary/technical school	210 (26.7%)
	College or university	173 (22.0%)
	Postgraduate education	33 (4.2%)
Self-rated health	1–5 scale	3.32 (0.75)
Previous arts participation	1–5 frequency scale	2.68 (1.03)

Values are presented as *n* (%) for categorical variables and *M* (SD) for continuous variables. Higher scores indicate better self-rated health and more frequent previous arts participation.

Baseline demographic comparability was examined across the four communities. No significant differences were observed in age-group distribution, *χ*^2^(9) = 8.23, *p* = 0.511, Cramér’s V = 0.059; gender, *χ*^2^(3) = 0.09, *p* = 0.993, Cramér’s V = 0.011; or educational attainment, *χ*^2^(12) = 15.24, *p* = 0.228, Cramér’s V = 0.080. Continuous age also did not differ significantly across sites, *F*(3, 782) = 0.07, *p* = 0.978, *η^2^* < 0.001. These findings indicated limited baseline demographic variation across the four community samples.

### 4.2 Measurement model

The measurement model demonstrated satisfactory reliability and convergent validity. As shown in Table 2, standardized indicator loadings ranged from 0.762 to 0.833. Cronbach’s alpha values ranged from 0.774 to 0.874, and McDonald’s omega values ranged from 0.774 to 0.873. Composite reliability values ranged from 0.855 to 0.909, while average variance extracted (AVE) values ranged from 0.596 to 0.686. All values met the recommended criteria for internal consistency and convergent validity (37, 38).

**Table 2:** Indicator loadings, reliability, and convergent validity.

Construct and indicator	Loading	Cronbach’s α	McDonald’s ω	CR	AVE
Perceived cultural appropriateness (CA)		0.847	0.847	0.897	0.686
The content of the performance was consistent with local cultural traditions.	0.823				
The stories and cultural elements were appropriate for this community.	0.83				
The form of participation was suitable for local residents.	0.833				
It was appropriate to conduct this immersive performance in this community.	0.826				
Performance co-creation engagement (PCE)		0.833	0.833	0.888	0.666
I actively participated in the interactive tasks.	0.818				
I contributed my own ideas, actions, or local stories.	0.81				
My participation helped shape the overall performance experience.	0.823				
I felt that I was a co-creator rather than only an audience member.	0.813				
Baseline social connectedness (SC-T0)		0.798	0.798	0.868	0.623
I feel connected to the people around me.	0.795				
I feel that I am part of this community.	0.762				
I can share experiences with other people in the community.	0.806				
I feel accepted when participating in community activities.	0.793				
Post-performance social connectedness (SC-T1)		0.774	0.774	0.855	0.596
I feel connected to the people around me.	0.779				
I feel that I am part of this community.	0.775				
I can share experiences with other people in the community.	0.77				
I feel accepted when participating in community activities.	0.765				
Baseline subjective vitality (VIT-T0)		0.874	0.874	0.909	0.666
At this moment, I feel energetic.	0.815				
At this moment, I feel alive and vital.	0.825				
At this moment, I feel alert and awake.	0.817				
At this moment, I have sufficient energy to engage in activities.	0.814				
At this moment, I feel psychologically active.	0.808				
Post-performance subjective vitality (VIT-T1)		0.872	0.873	0.907	0.662
At this moment, I feel energetic.	0.805				
At this moment, I feel alive and vital.	0.821				
At this moment, I feel alert and awake.	0.827				
At this moment, I have sufficient energy to engage in activities.	0.816				
At this moment, I feel psychologically active.	0.799				

α, Cronbach’s alpha; ω, McDonald’s omega; CR, composite reliability; AVE, average variance extracted; T0, pre-performance measurement; T1, post-performance measurement.

Discriminant validity was assessed using the Fornell–Larcker criterion and the Heterotrait–Monotrait ratio (HTMT). As reported in Table 3, the square root of the AVE for each construct exceeded its correlations with the other constructs, and all HTMT values were below 0.85 (39). The highest HTMT value was 0.770 between SC-T0 and SC-T1, supporting adequate discriminant validity. The descriptive statistics and correlations among the study constructs are also presented in Table 3.

**Table 3:** Descriptive statistics, correlations, and discriminant validity.

Construct	1	2	3	4	5	6	M	SD
1. CA	**0.828**	0.677	0.053	0.303	0.037	0.225	5.34	0.82
2. PCE	0.569	**0.816**	0.059	0.441	0.034	0.326	5.04	0.87
3. SC-T0	−0.030	−0.017	**0.789**	0.770	0.309	0.569	3.27	0.63
4. SC-T1	0.246	0.354	0.606	**0.772**	0.247	0.695	3.48	0.58
5. VIT-T0	0.009	0.001	0.258	0.203	**0.816**	0.696	4.20	0.93
6. VIT-T1	0.194	0.278	0.475	0.571	0.608	**0.814**	4.57	0.88

Diagonal values in bold represent the square root of the AVE. Values below the diagonal are construct correlations, and values above the diagonal are HTMT ratios. CA, perceived cultural appropriateness; PCE, performance co-creation engagement; SC, social connectedness; VIT, subjective vitality.

Because several variables were obtained through self-report at the same post-performance measurement occasion, common-method variance was also considered. Harman’s single-factor test indicated that the first unrotated factor accounted for 34.4% of the total variance. This result was treated only as a descriptive diagnostic and does not rule out common-method variance, particularly for CA, PCE, SC-T1, and VIT-T1, which were assessed from the same respondents at the same occasion.

### 4.3 Longitudinal measurement invariance

Longitudinal measurement invariance was examined separately for social connectedness and subjective vitality. As shown in Table 4, the configural model for social connectedness demonstrated good fit, *χ*^2^(15) = 19.33, CFI = 0.998, TLI = 0.996, RMSEA = 0.019, and SRMR = 0.017. The metric model also showed adequate fit, *χ*^2^(18) = 23.38, CFI = 0.997, TLI = 0.996, RMSEA = 0.020, and SRMR = 0.026, with changes in fit indices remaining within the prespecified criteria (ΔCFI = −0.001, ΔRMSEA = 0.000, ΔSRMR = 0.009). Further constraining item intercepts produced a well-fitting scalar model, *χ*^2^(21) = 23.42, CFI = 0.999, TLI = 0.998, RMSEA = 0.012, and SRMR = 0.026 (ΔCFI = 0.001, ΔRMSEA = −0.007, ΔSRMR = 0.000).

**Table 4:** Longitudinal measurement invariance of social connectedness and subjective vitality.

Construct	Model	*χ*^2^ (df)	CFI	TLI	RMSEA	SRMR	ΔCFI	ΔRMSEA	ΔSRMR
Social connectedness	Configural	19.33 (15)	0.998	0.996	0.019	0.017	—	—	—
	Metric	23.38 (18)	0.997	0.996	0.020	0.026	−0.001	0.000	0.009
	Scalar	23.42 (21)	0.999	0.998	0.012	0.026	0.001	−0.007	0.000
Subjective vitality	Configural	26.83 (29)	1.000	1.000	0.000	0.013	—	—	—
	Metric	29.28 (33)	1.000	1.000	0.000	0.019	0.000	0.000	0.006
	Scalar	32.89 (37)	1.000	1.000	0.000	0.020	0.000	0.000	0.000

A similar pattern was observed for subjective vitality. The configural model showed good fit, *χ*^2^(29) = 26.83, CFI = 1.000, TLI = 1.000, RMSEA = 0.000, and SRMR = 0.013. Both the metric model, *χ*^2^(33) = 29.28, CFI = 1.000, TLI = 1.000, RMSEA = 0.000, SRMR = 0.019, and the scalar model, *χ*^2^(37) = 32.89, CFI = 1.000, TLI = 1.000, RMSEA = 0.000, SRMR = 0.020, met the invariance criteria. Changes in fit were minimal for the metric model (ΔCFI = 0.000, ΔRMSEA = 0.000, ΔSRMR = 0.006) and scalar model (ΔCFI = 0.000, ΔRMSEA = 0.000, ΔSRMR = 0.000). These results supported longitudinal scalar invariance for both constructs, providing a basis for the subsequent pre–post comparisons.

### 4.4 Pre–post changes in social connectedness and subjective vitality

Following establishment of longitudinal scalar invariance for both constructs, paired-samples comparisons were conducted to examine pre–post differences in social connectedness and subjective vitality. Social connectedness was higher after the performance (*M* = 3.48, SD = 0.58) than at baseline (*M* = 3.27, SD = 0.63), with a mean difference of 0.216, 95% CI [0.178, 0.254], *t*(785) = 11.18, *p* < 0.001, *d*z = 0.40. H1 was therefore supported (see Table 5).

**Table 5:** Pre–post comparisons of social connectedness and subjective vitality.

Outcome	Pre-performance *M* (SD)	Post-performance *M* (SD)	Mean change	95% CI for change	*t*	*d*z	Result
Social connectedness	3.27 (0.63)	3.48 (0.58)	0.216	[0.178, 0.254]	11.18***	0.40	H1 supported
Subjective vitality	4.20 (0.93)	4.57 (0.88)	0.370	[0.314, 0.426]	12.93***	0.46	H2 supported

*N* = 786. dz represents the standardized within-participant mean difference. ***p < 0.001.

Subjective vitality was also higher after the performance (*M* = 4.57, SD = 0.88) than at baseline (*M* = 4.20, SD = 0.93). The mean difference was 0.370, 95% CI [0.314, 0.426], *t*(785) = 12.93, *p* < 0.001, *d*z = 0.46. H2 was therefore supported.

### 4.5 Structural model and indirect associations

Variance inflation factor values ranged from 1.00 to 1.66, indicating no substantial multicollinearity in the structural model. The model explained 35.2% of the variance in performance co-creation engagement, 50.4% of the variance in post-performance social connectedness, and 59.5% of the variance in post-performance subjective vitality. The corresponding cross-validated Q^2^ values were 0.336, 0.491, and 0.580, respectively.

Perceived cultural appropriateness was positively associated with performance co-creation engagement (*β* = 0.551, *p* < 0.001), supporting H3. Performance co-creation engagement was positively associated with post-performance social connectedness after adjustment for baseline social connectedness and the specified covariates (*β* = 0.355, *p* < 0.001), supporting H4. Post-performance social connectedness was positively associated with post-performance subjective vitality after adjustment for baseline vitality and the other model variables (*β* = 0.419, *p* < 0.001), supporting H5. Baseline social connectedness and baseline subjective vitality were also positively associated with their respective post-performance measures (*β* = 0.612 and 0.522, respectively, both *p* < 0.001). In addition, performance co-creation engagement showed a significant positive association with post-performance subjective vitality (*β* = 0.123, *p* < 0.001).

Bootstrap analyses indicated two significant indirect associations between perceived cultural appropriateness and post-performance subjective vitality. The specific indirect association through performance co-creation engagement was significant (*β* = 0.068, SE = 0.016, 95% BC CI [0.037, 0.102]), as was the hypothesized serial indirect association through performance co-creation engagement and post-performance social connectedness (*β* = 0.082, SE = 0.009, 95% BC CI [0.065, 0.100]), supporting H6. The total indirect association was *β* = 0.149 (SE = 0.018, 95% BC CI [0.115, 0.189]). The direct association between perceived cultural appropriateness and post-performance subjective vitality was not statistically significant (*β* = 0.017, SE = 0.029, 95% BC CI [−0.038, 0.076], *p* = 0.570), whereas the total association was significant (*β* = 0.167, SE = 0.026, 95% BC CI [0.119, 0.217]).

Overall, the findings indicated multiple indirect associations rather than a single indirect-only pathway. Because perceived cultural appropriateness, performance co-creation engagement, post-performance social connectedness, and post-performance subjective vitality were assessed at the same post-performance occasion, these estimates are interpreted as theory-informed, baseline-adjusted structural associations rather than evidence of temporally established or causal mediation (see Table 6).

**Table 6:** Structural model estimates and indirect associations.

Effect	*β*	SE	95% bootstrap CI	*p*	Result
Panel A. Structural associations					
H3: Association of CA with PCE	0.551	0.026	[0.499, 0.601]	<0.001	Supported
H4: Association of PCE with SC-T1	0.355	0.026	[0.306, 0.405]	<0.001	Supported
H5: Association of SC-T1 with VIT-T1	0.419	0.025	[0.371, 0.468]	<0.001	Supported
Baseline association of SC-T0 with SC-T1	0.612	0.023	[0.566, 0.656]	<0.001	—
Baseline association of VIT-T0 with VIT-T1	0.522	0.025	[0.473, 0.570]	<0.001	—
Supplementary association of PCE with VIT-T1	0.123	0.029	[0.065, 0.178]	<0.001	—
Panel B. Indirect, direct, and total associations					
Specific indirect association of CA with VIT-T1 through PCE	0.068	0.016	[0.037, 0.102]	<0.001	—
H6: Serial indirect association of CA with VIT-T1 through PCE and SC-T1	0.082	0.009	[0.065, 0.100]	<0.001	Supported
Total indirect association	0.149	0.018	[0.115, 0.189]	<0.001	—
Direct association of CA with VIT-T1	0.017	0.029	[−0.038, 0.076]	0.57	—
Total association of CA with VIT-T1	0.167	0.026	[0.119, 0.217]	<0.001	—

### 4.6 Community sensitivity analyses

Site-specific analyses showed positive pre–post changes in both social connectedness and subjective vitality across all four communities. Mean changes in social connectedness ranged from 0.191 to 0.244, while mean changes in subjective vitality ranged from 0.334 to 0.458. Leave-one-community-out analyses produced similar estimates for the hypothesized serial indirect association (*β* = 0.079–0.086), suggesting that the overall structural pattern was not driven primarily by any single community.

## 5 Discussion

### 5.1 Summary and interpretation of main findings

Participants reported higher social connectedness and subjective vitality immediately after the immersive performances than at baseline, with small-to-moderate within-participant differences. These findings are broadly consistent with research suggesting that participatory arts can provide opportunities for social interaction, shared experience, and positive psychosocial engagement (2, 10, 13, 14). The participatory features of the performances, including role-taking, collective storytelling, movement, and collaborative tasks, may have created conditions conducive to interpersonal connection and psychological activation. However, subjective vitality should be interpreted as an immediate state-like experience of energy and psychological aliveness rather than as a broader improvement in mental health or general wellbeing (31).

The structural findings were also consistent with the proposed theoretical framework. Higher perceived cultural appropriateness was associated with stronger performance co-creation engagement, suggesting that locally meaningful stories, dialects, objects, and community memories may provide participants with relevant cultural resources for active participation (16, 21). Greater co-creation engagement was, in turn, associated with stronger post-performance social connectedness, consistent with social identity perspectives and research showing that shared and coordinated activities can facilitate interpersonal bonding (27–30). Importantly, the analysis indicated two significant indirect associations between cultural appropriateness and post-performance vitality: one through co-creation engagement alone and another through co-creation engagement and social connectedness. This pattern suggests that active participation may be associated with vitality both directly and through participants’ social experience, consistent with the INNATE framework’s emphasis on interacting programme, experiential, and contextual ingredients (15).

These findings should nevertheless be interpreted cautiously. The nonsignificant direct association between cultural appropriateness and post-performance subjective vitality does not indicate that cultural appropriateness operates exclusively through co-creation or social connectedness. Moreover, cultural appropriateness, co-creation engagement, post-performance social connectedness, and post-performance subjective vitality were assessed at the same post-performance occasion. Because mediation implies an underlying temporal process, statistical ordering among concurrently measured variables does not by itself establish temporal precedence or causal mediation (44–47). More rigorous mediation inference generally requires temporal separation of the proposed process variables and stronger control of alternative explanations (48, 49). Accordingly, the present structural findings are best interpreted as theory-informed, baseline-adjusted associations that are consistent with the proposed indirect pathways rather than as evidence of a sequential causal mechanism.

### 5.2 Theoretical contributions

First, this study extends arts-and-health research by examining co-created immersive performance as a community-based cultural activity in rural and peri-rural China. Previous research has focused more frequently on museums, music, visual arts, dance, and arts-prescribing programmes, while immersive performance remains comparatively underexplored in community health research (2, 6, 33). By combining spatial participation, role-taking, local storytelling, and collective decision-making, immersive performance provides a distinctive form of active arts engagement that connects participatory performance scholarship with arts-and-health research (19, 20). The findings therefore broaden the range of cultural activities that may be relevant to social participation and immediate psychosocial experience.

Second, the study contributes an integrative theoretical framework linking cultural appropriateness, co-creation engagement, social connectedness, and subjective vitality. Rather than treating artistic participation as a uniform exposure, the framework distinguishes culturally meaningful programme features from participants’ experiential engagement and subsequent social and psychological states. This distinction is consistent with the INNATE framework, which emphasizes interactions among programme characteristics, participant experiences, and contextual conditions in arts-in-health activities (15), and with cultural adaptation research highlighting the importance of perceived relevance and appropriateness (16). The observed structural pattern was consistent with this proposed ordering, although it should be understood as a theory-informed association framework rather than as evidence of an established causal mechanism.

Third, the study extends co-creation perspectives by conceptualizing residents’ cultural knowledge, memories, actions, and decisions as resources in both artistic participation and social interaction (21–23). In this context, shared cultural memory may provide a common resource through which older and younger residents contribute to and reinterpret local narratives, while role-taking and coordinated participation may strengthen a temporary sense of shared group membership. This interpretation connects co-creation with social identity perspectives, which emphasize the importance of shared group participation for belonging and social connection (27, 28). Evidence that coordinated artistic activities can facilitate rapid interpersonal bonding further supports this conceptual link (29, 30). Because social identity and intergenerational cultural transmission were not directly measured, however, they should be regarded as theoretical extensions for future testing rather than mechanisms established by the present data.

### 5.3 Implications for community health practice

The findings suggest that community-based immersive performances should emphasize cultural relevance and meaningful participation rather than artistic exposure alone. Programme teams can adapt local stories, dialects, objects, music, and performance spaces in consultation with residents and local cultural actors, while providing accessible opportunities for story sharing, role interaction, collective movement, and narrative decision-making. Such an approach is consistent with cultural adaptation and co-creation perspectives, which emphasize perceived relevance, participant contribution, and contextual responsiveness (15, 16, 23, 36). Evaluation should therefore consider not only attendance and satisfaction but also the quality of participation and opportunities for social interaction.

In rural Chinese settings, village committees, rural cultural stations, community workers, and trusted local cultural knowledge holders may also function as an informal referral architecture for connecting residents with non-clinical cultural activities. Rather than reproducing healthcare-based social prescribing models directly, these locally embedded structures could disseminate information, identify suitable community activities, encourage participation, and connect residents with cultural and social resources. This community-oriented approach is consistent with broader social prescribing literature emphasizing the importance of local assets, referral relationships, and context-sensitive implementation (1, 3, 4). Such arrangements may be particularly relevant where formal link-worker systems are not well established.

At the municipal level, health authorities could explore partnerships with cultural agencies, community cultural stations, and local organizations to pilot culturally adapted participatory arts activities within active-ageing and rural community health-promotion programmes. For example, periodic immersive performances could be incorporated into community activity schedules alongside initiatives aimed at increasing social participation and reducing isolation among older residents. Because social relationships are an important component of health and healthy ageing, programme monitoring could include participation, social connectedness, and short-term subjective vitality alongside implementation indicators (13, 25, 28, 33). Given the present study design, however, such applications should initially be treated as pilot community-health strategies whose longer-term effectiveness requires evaluation through controlled and longitudinal research.

### 5.4 Limitations and future research directions

Several limitations should be acknowledged. First, the single-group pre–post design did not include a comparison condition, so the observed changes cannot be attributed definitively to the performances. In addition, cultural appropriateness, co-creation engagement, post-performance social connectedness, and post-performance vitality were assessed at the same occasion. Their statistical ordering therefore does not establish temporal precedence or causal mediation (44, 46). Future studies should use controlled longitudinal designs with temporally separated assessments of the proposed process variables.

Second, outcomes were assessed immediately after participation and relied primarily on self-report. The observed difference in subjective vitality may partly reflect short-lived emotional or physical activation, and common-method or socially desirable responding cannot be fully excluded (31, 43). Future research should incorporate behavioral or observational measures and assess whether changes persist at follow-up points such as 2 weeks and 1 month after participation.

Finally, participants were recruited voluntarily from only four communities in Guangzhou. Although community site was controlled and sensitivity analyses indicated similar patterns across sites, four clusters are insufficient to fully address community-level dependence. Future studies should include a larger number of communities, use multilevel or cluster-based designs where appropriate, and replicate the findings across more diverse populations and settings (50, 51).

## Data Availability

The original contributions presented in the study are included in the article/supplementary material, further inquiries can be directed to the corresponding author.
